# Evaluating glycolysis-associated biomarkers for radiotherapy sensitivity in head and neck squamous cancer

**DOI:** 10.3389/fimmu.2026.1736778

**Published:** 2026-06-29

**Authors:** Bilan Xie, Hainan Yang, Chunyuan Lin, Jinzhi Lai

**Affiliations:** 1 Department of Oncology, The Second Affiliated Hospital of Fujian Medical University, Quanzhou, Fujian, China; 2 Department of Ultrasound, First Affiliated Hospital of Xiamen University, School of Medicine, Xiamen University, Xiamen, Fujian, China

**Keywords:** glycolysis, head and neck squamous cell carcinoma, radiosensitivity, radiotherapy, tumor immune microenvironment

## Abstract

**Background:**

Head and neck squamous cell carcinoma (HNSC) present a significant treatment challenge due to variability in radiotherapy response, where glycolysis may play a pivotal role in modulating sensitivity. This study aimed to develop a glycolysis-associated biomarker for radiotherapy sensitivity.

**Methods:**

We analyzed gene expression and clinical data from 491 HNSC patients and single-cell RNA sequencing to assess glycolysis activity in HNSC. A glycolysis-associated radiosensitivity index (RI) was developed using Cox regression analysis of glycolysis-related genes. Immune microenvironment profiles, functional pathways, and therapeutic responses were analyzed via using ssGSEA, CIBERSORT, TIDE, and pRRophetic. *In vitro* experiments validated glycolytic activity and radiosensitivity in HNSC cell lines.

**Results:**

The study identified that low glycolytic activity significantly correlates with improved overall survival in HNSC patients following radiotherapy, while high glycolytic activity is associated with radio-resistance. Using a RI based on glycolysis-related gene expression, we successfully stratified patients into RS and RR groups. RS tumors exhibited significantly higher immune cell infiltration and lower TIDE scores, indicating a better response to immunotherapy. In contrast, RR tumors showed increased sensitivity to chemotherapy agents, including platinum and EGFR/HER2 inhibitors. Single-cell RNA sequencing revealed that high-glycolysis tumors had diminished immune cell infiltration, particularly lacking CD8+ T cells, supporting the notion of immune evasion. *In vitro* validation further demonstrated that radioresistant CAL-27IR cells displayed elevated glycolytic activity and upregulation of key genes associated with the RI, showing the link between glycolysis and radiation resistance.

**Conclusion:**

This study emphasizes the importance of glycolytic activity in influencing radiotherapy sensitivity and highlights the potential of the RI as a predictive biomarker, paving the way for improved personalized treatment strategies in HNSC.

## Introduction

Head and neck squamous cell carcinoma (HNSC) represents a significant global health challenge, with increasing incidence rates over the past few decades. Accounting for more than 900, 000 new cases diagnosed annually and a significant impact on public health (1). While radiotherapy remains a cornerstone of treatment, particularly for localized disease, resistance to radiation presents a major challenge (2). Up to 30% of patients with HNSC may exhibit primary or acquired radio-resistance, complicating treatment outcomes and leading to higher rates of local recurrence (3). The differences in results, along with the chance of serious side effects, lead to an important question: how can we better predict which patients will benefit from radiotherapy and which ones might need other treatment options? Thus, addressing this challenge of predicting radiotherapy sensitivity represents a central unmet need in HNSC clinical practice.

Glycolysis, a metabolic pathway central to tumor survival, has emerged as a key player in HNSC progression and therapeutic resistance (4). Tumors often exhibit increased glycolytic activity, a phenomenon known as the Warburg effect, which allows for rapid energy production and supports pathways essential for growth and proliferation (5). In HNSC, glycolysis has been linked to tumor progression and metastasis, reflecting a complex interplay between metabolic reprogramming and tumor behavior (6). Intriguingly, the relationship between glycolysis and radiotherapy is complex and not fully understood (7). Some studies suggest that increased glycolysis can enhance radiosensitivity in certain contexts, while other research suggests that elevated glycolysis may contribute to radio-resistance through mechanisms involving hypoxia (8). Given these associations, a more refined understanding of the interplay between glycolysis and radiotherapy response is warranted, potentially allowing us to leverage glycolytic activity as a predictive marker for predicting radiotherapy susceptibility in patients with HNSC (9).

With advancements in high-throughput omics technologies, the ability to analyze tumor gene profiles has transformed our understanding of cancer biology and treatment responses (10, 11). Various studies have employed genomic and transcriptomic approaches to develop predictive models for therapeutic outcomes, including radiotherapy (12). For instance, one study established a prognostic signature based on ten genes, which showed consistent prognostic relevance across numerous cancer cell lines (13). Parallel to this, a more extensive 31-gene expression profile has been developed, offering a specialized means to gauge how tumors are likely to respond to radiotherapy (14). However, few studies have specifically utilized the capability of high-throughput analyses to explore the role of glycolysis in predicting radiotherapy response in HNSC. Given the relationship between metabolic pathways and radiation sensitivity, there is significant potential to exploit glycolysis-related gene model for improving therapeutic predictions in this context.

Here, we present a comprehensive analysis of the relationship between glycolysis-related gene expression and radiotherapy sensitivity in patients with HNSC. By integrating data from publicly available databases, single-cell RNA sequencing, and *in vitro* experiments, we developed and validated a radiosensitivity index (RI) based on the expression of key glycolysis-related genes. Our findings indicate a significant association between glycolysis scores and therapeutic outcomes, providing insights into potential mechanisms underlying treatment resistance. Our findings highlight the potential of the glycolysis-based RI as a predictive biomarker for radiotherapy outcomes in HNSC, paving the way for more personalized and effective treatment strategies.

## Materials and methods

### Patient cohort and data acquisition

To investigate the role of glycolysis in HNSC, gene expression and clinical data were accessed via the UCSC Xena platform (https://xena.ucsc.edu/) (15). The initial data, provided in Fragments Per Kilobase Million (FPKM) units, were transformed into Transcripts Per Million (TPM) to normalize for transcript length and sequencing depth. Inclusion criteria were implemented to ensure the quality and homogeneity of the dataset. Only primary HNSC tumor samples were considered. Furthermore, patients were required to possess complete clinical follow-up information extending beyond 30 days post-diagnosis. Detailed information on radiotherapy treatment was also a prerequisite for inclusion. Ultimately, a cohort of 491 patients was identified and selected for subsequent analyses, each accompanied by corresponding RNA sequencing data and comprehensive clinical information.

A curated list of 289 genes involved in glycolysis was compiled based on the HALLMARK_GLYCOLYSIS and KEGG GLYCOLYSIS GLUCONEOGENESIS gene sets obtained from the MsigDB database (16). To determine the level of glycolytic activity in each tumor sample, single-sample Gene Set Enrichment Analysis (ssGSEA) was performed (17). This method allows for the quantification of pathway enrichment at the individual sample level, providing a glycolysis activity score for each patient. Based on the median ssGSEA score within the study cohort, patients were then stratified into subgroups exhibiting either high or low glycolytic activity for further comparative analyses.

### Single-cell RNA sequencing data processing and glycolytic activity assessment

Single-cell RNA sequencing data from HNSC samples (GSE103322) were obtained from the TISCH2 database(http://tisch.comp-genomics.org/) (18, 19). Specifically, our analysis was restricted to cells derived from primary tumor samples The “single cell expression matrix” file was processed using the Seurat package (v4) in R (v4.3) (20). The data were normalized using the NormalizeData function, and highly variable genes were identified using the Find Variable Features function. Principal component analysis (PCA) was performed on the scaled data, followed by dimensionality reduction using UMAP and t-SNE. Cell clusters were identified using the FindClusters function. Cell type annotation was performed based on known marker genes from the TISCH2 and CellMarker databases, using the FindAllMarkers function to identify cluster-specific genes (21). Glycolytic activity scores were calculated for each cell using the AUCell package (v1.18) and the HALLMARK glycolysis gene set (22). Cells were then divided into high- and low-glycolysis groups based on the median glycolysis score. Differential expression of glycolysis-related genes was analyzed across cell types. The ggplot2 package was used for data visualization.

### Development of the radiosensitivity index using glycolysis-related genes

To construct a clinically applicable predictive model from the broader metabolic profile, we filtered the aforementioned 289 glycolysis-related genes to identify the most critical prognostic candidates. Specifically, to investigate the relationship between glycolysis-related gene expression and overall survival (OS) in patients undergoing radiotherapy, we first performed univariate Cox regression analysis. Subsequently, a multivariate Cox regression analysis was undertaken to further refine the selection of glycolysis-related prognostic genes, thereby constructing a radiosensitivity index. This index was formulated using the expression levels of identified genes in conjunction with their respective regression coefficients to derive a RI for each individual patient, calculated as follows:


$$\text{Radiosensitivity index}=\sum_{n=1}^{n}(Expression\;of\;Gene\;i\times Coefficient\;of\;Gene\;i)$$

where *n* represents the number of selected genes. Patients were then stratified into two subgroups based on the median RI value: the radiosensitive (RS) subgroup and the radioresistant (RR) subgroup. The RS group consists of patients expected to exhibit enhanced OS due to radiotherapy relative to their counterparts who did not receive treatment. Conversely, the RR group includes patients who showed no significant survival advantage from radiotherapy, regardless of their treatment status. To identify differentially expressed genes (DEGs) between the RS and RR subgroups, the “limma” package was utilized. DEGs were defined based on the criteria of a false discovery rate (FDR)< 0.01 and |log2(fold change)| > 1.

### Analysis of functional pathways and immune cell infiltration

To explore the variations in cellular pathways between the RS and RR groups, we employed Gene Set Variation Analysis (GSVA) for the enrichment analysis of HALLMARK pathways (23). This approach enabled us to identify the molecular pathways that were significantly enriched within each group. Additionally, we performed Gene Ontology (GO) enrichment analysis to gain a more comprehensive understanding of the biological processes (BP), molecular functions (MF), and cellular components (CC) pertinent to each group. These analyses clarified the functional distinctions that underpin differential radiosensitivity in HNSC.

To examine the tumor immune microenvironment and assess immune cell infiltration patterns in both RS and RR groups, we employed the ESTIMATE algorithm to estimate the relative abundance of infiltrating immune and stromal cells (24). This algorithm generates three distinct scores: the immune score, stromal score, and the integrated ESTIMATE score. These scores provide measures of the relative abundance of immune and stromal cell types within the tumor microenvironment. Additionally, the CIBERSORT computational method was applied to deconvolute the composition of tumor-infiltrating immune cells, enabling the estimation of proportions for 22 unique immune cell subtypes (25). Only samples that yielded a CIBERSORT-derived p-value of less than 0.05 were included in subsequent analyses. Resulting immune cell fractions were normalized to ensure their sum equaled one, permitting proportional comparisons across samples.

### Prediction of responses to immunotherapy, chemotherapy, and targeted therapy

In order to assess the potential of HNSC patients to respond favorably to immunotherapy interventions, we calculated Tumor Immune Dysfunction and Exclusion (TIDE) scores. These scores were obtained through the TIDE portal (http://tide.dfci.harvard.edu/) (26), which combines transcriptomic data to produce TIDE scores as well as separate measurements for T cell dysfunction and T cell exclusion. Together, these scores offer an indication of the likelihood of resistance or sensitivity to immune checkpoint blockade.

For the evaluation of responses to conventional chemotherapy and targeted treatments, we utilized the pRRophetic R package, a tool designed to predict drug sensitivity based on gene expression profiles (27). This package leverages baseline sensitivity data derived from the Genomics of Drug Sensitivity in Cancer (GDSC) database to estimate the half-maximal inhibitory concentration (IC_50_) values for various commonly utilized anticancer agents. This predictive capability facilitates the stratification of patients based on their expected therapeutic response.

### Development of radioresistant HNSC cells

The human tongue squamous cell carcinoma (TSCC) cell line CAL-27 was procured from the China Center for Type Culture Collection (CCTCC, Wuhan, China). For cell maintenance, the cells were grown in RPMI 1640 medium (Corning, United States) supplemented with 10% fetal bovine serum (Corning, United States) and 1% antibiotic solution (Gibco-BRL, Gaithersburg, MD, United States). Cultures were kept at 37 °C in a humidified incubator with an atmosphere containing 5% CO2. Prior to any experimental procedures, mycoplasma contamination was assessed and ruled out. To generate a radioresistant cell line, designated CAL-27IR, CAL-27 cells were exposed to a series of escalating radiation doses (2, 4, 6, 8, 10, 10, and 10 Gy) over seven treatment cycles, culminating in a cumulative dose of 50 Gy (28). The radio-resistance of the CAL-27IR cell line was subsequently assessed through the Cell Counting Kit-8 (CCK-8) assay. Cell viability of CAL-27 and CAL-27IR cells was measured at hours following radiation. In this procedure, both CAL-27IR and CAL-27 cells were plated in 96-well plates and exposed to different radiation doses. After irradiation, cells were treated with CCK-8 solution, and the extent of cell viability was quantified by measuring the optical density (OD) at 450 nm using a microplate reader. The OD450 measurements were found to correlate directly with cell viability.

### Quantitative real-time polymerase chain reaction

Total RNA was extracted from the cultured cells utilizing TRIzol^®^ reagent (Invitrogen, San Diego, CA, USA), following the protocols provided by the manufacturer. The concentration and purity of the extracted RNA were assessed using a NanoDrop 2000 spectrophotometer (Thermo Fisher Scientific, USA) to ensure that the RNA was suitable for downstream applications.

Gene expression analysis was performed through qRT-PCR with the SYBR^®^ PrimeScript™ RT-PCR Kit (Invitrogen, USA) in accordance with the manufacturer’s instructions. The specific primer sequences used for amplification are listed in **Supplementary Table 1**. Each qRT-PCR reaction was conducted in triplicate, accompanied by no-template controls to verify the absence of contamination or non-specific amplification. Threshold cycle (Ct) values were normalized against the average Ct values of reference genes, with GAPDH serving as the internal standard. The relative expression levels of target mRNAs were calculated using the 2^^−ΔΔCt^ method, with results presented as fold change relative to the control samples.

### Assessment of cellular glycolytic capacity

To determine the glycolytic capacity of the cells, both the extracellular acidification rate (ECAR) and the oxygen consumption rate (OCR) were quantified using the XF96 Extracellular Flux Analyzer (Seahorse Bioscience). All media and reagents required for these measurements were prepared in accordance with the guidelines provided by the manufacturer. CAL-27 and CAL-27IR cells were plated in XF96 cell culture microplates at a density of 5 × 10^4^ cells per well. After allowing for an overnight attachment, the growth medium was removed and replaced with fresh assay medium containing designated metabolic modulators. Subsequent measurements of ECAR and OCR were conducted utilizing the Seahorse XF Glycolytic Rate Assay Kit and the Seahorse XF Cell Mito Stress Test Kit (Seahorse Bioscience), following the provided protocols. To adjust for any possible differences in cell density, ECAR and OCR values were normalized to the cell count within each well.

### Statistical analysis

Statistical analyses were conducted using R software (version 4.1.3). The choice of statistical tests was determined by the nature and distribution of the data involved. For comparisons of categorical variables or pairwise characteristics among different groups, the Chi-square test was employed. When examining differences between two independent groups, the Mann-Whitney U test was utilized to determine statistical significance. For the comparison of categorical variables across multiple independent groups, the Kruskal-Wallis test was applied. To assess the presence of linear relationships among normally distributed variables, Pearson’s correlation coefficient was computed. For non-parametric data exhibiting non-normal distributions, Spearman’s rank correlation coefficient was used to analyze correlations. Survival outcomes among groups were evaluated using Kaplan-Meier survival curves, with differences tested for significance through the log-rank test. Unless specified otherwise, all statistical hypothesis tests were conducted as two-sided, and a p-value of less than 0.05 was regarded as indicative of statistical significance. This threshold was uniformly applied across all analyses to ensure consistency and clarity in interpretation.

## Results

### Glycolysis activity and radiotherapy efficacy in HNSC

**Figure 1:**
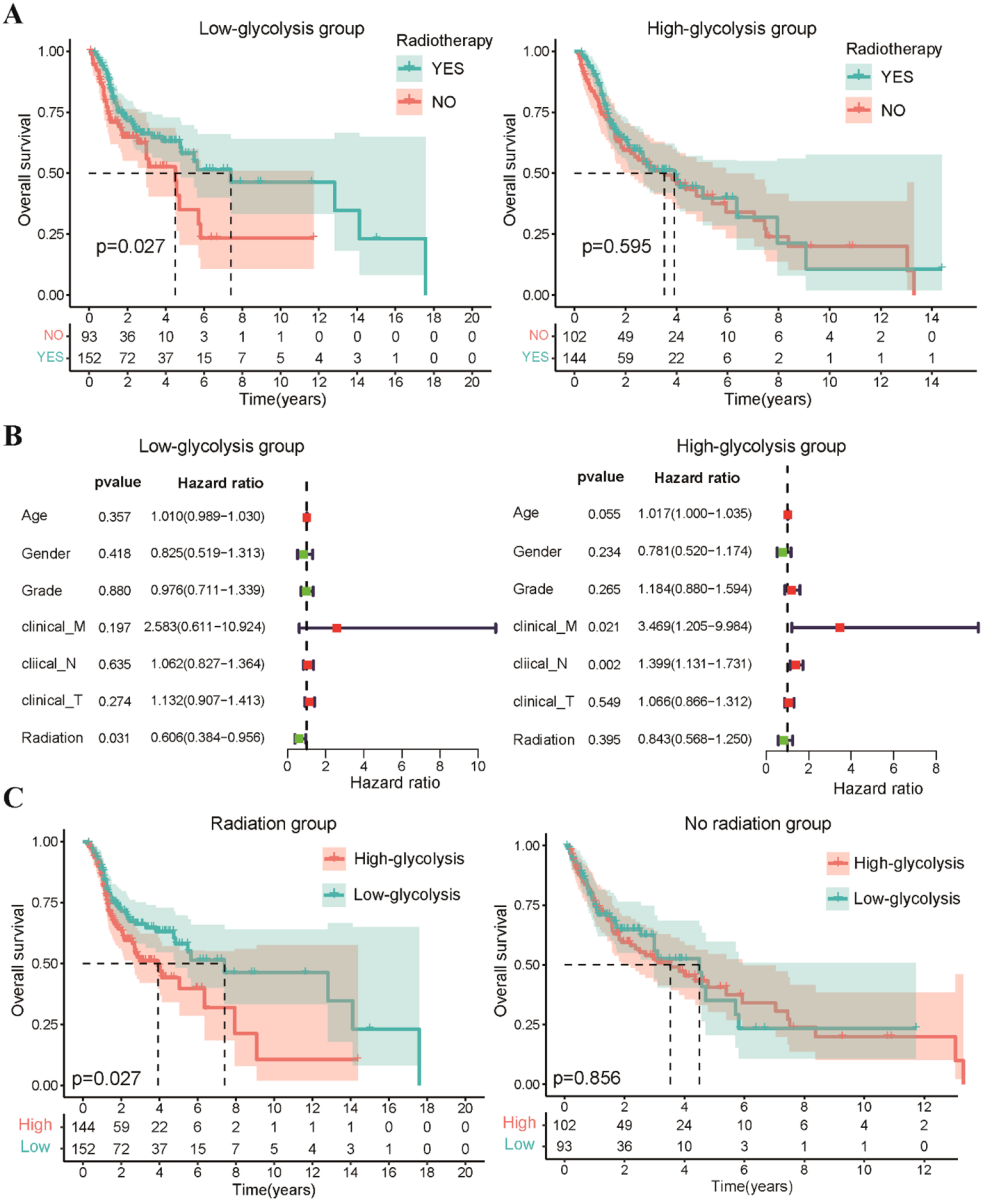
Relationships between glycolysis activity and radiotherapy outcomes in HNSC patients. **(A)** Kaplan-Meier survival curves illustrate a significant survival advantage for low glycolysis patients receiving radiotherapy compared to those who did not, unlike the high glycolysis group. **(B)** Multivariate Cox regression analysis assessing the impact of radiation and clinical variables in both low and high glycolysis groups. **(C)** Overall survival curves comparing patients receiving and not receiving radiotherapy within both low and high glycolysis groups.

This study investigates the impact of glycolysis on the effectiveness of radiotherapy in patients diagnosed with HNSC. A total of 289 glycolysis-related genes were acquired from the KEGG and MSigDB databases, allowing us to stratify patients into low and high glycolysis score categories using the ssGSEA algorithm. Results indicated distinct outcomes in radiotherapy based on glycolysis levels. Specifically, patients in the low-glycolysis cohort demonstrated a marked improvement in OS when compared to those who did not receive radiotherapy. Conversely, among the high-glycolysis cohort, the difference in survival rates between radiotherapy recipients and non-recipients was not statistically significant (**Figure 1A**). Conducting a multivariate Cox regression analysis, we found that radiation served as an independent prognostic factor in the low-glycolysis group, while this association was absent in the high-glycolysis cohort (**Figure 1B**). To further clarify this relationship, we directly compared the survival outcomes of high and low glycolysis groups within the treated cohorts. Among patients who received radiotherapy, those in the low-glycolysis group exhibited a significantly better OS compared to the high-glycolysis group. In contrast, no significant survival difference was observed between the two groups in the non-radiotherapy cohort (**Figure 1C**). These findings suggest that glycolytic activity plays a crucial role in determining the sensitivity of HNSC tumors to radiotherapy.

### Glycolysis activity and immune cell infiltration in HNSC

**Figure 2:**
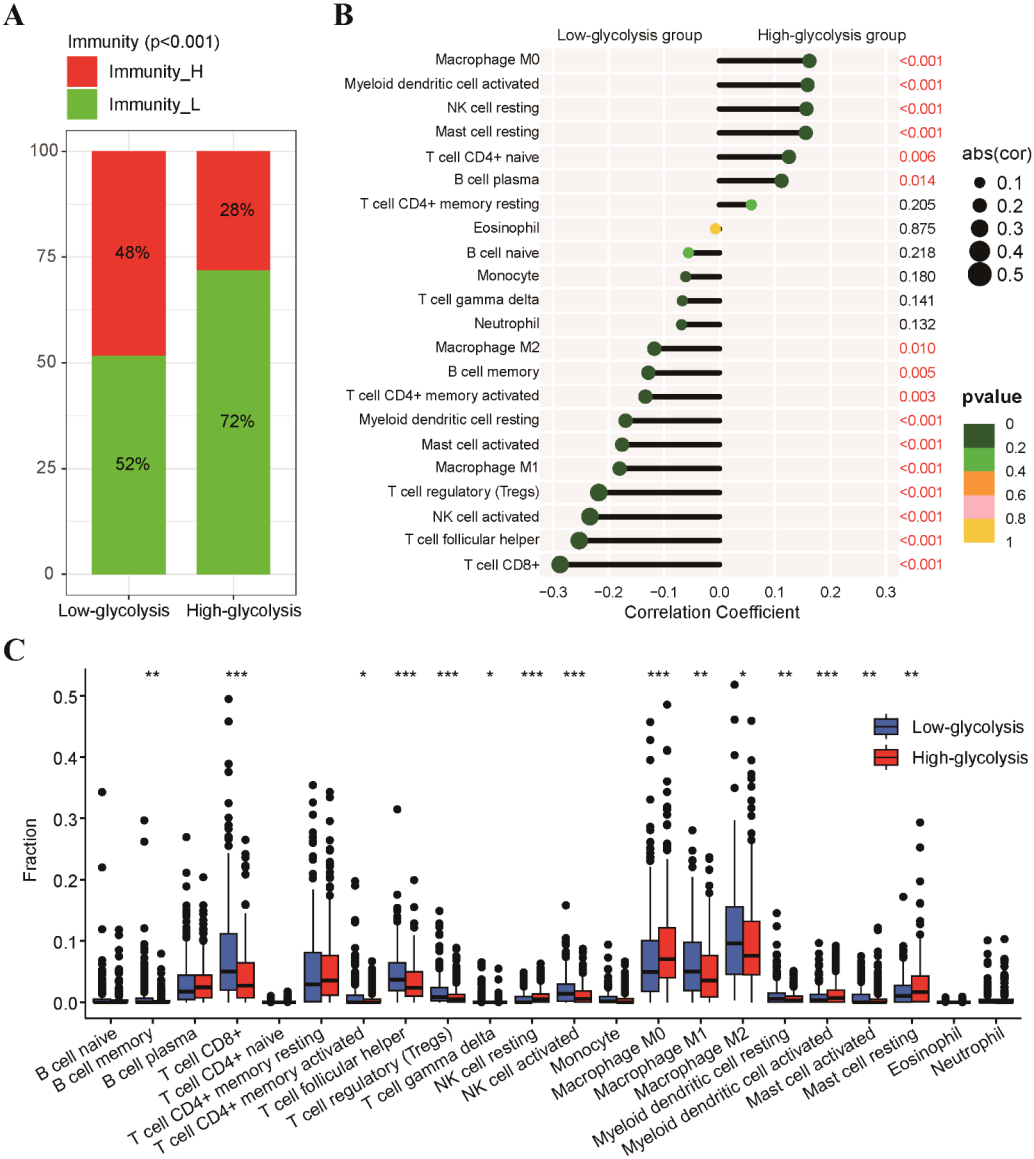
Influence of glycolytic levels on immune cell infiltration in patients with HNSC. **(A)** A stacked histogram displaying the proportions of patients with high immunity versus low immunity across glycolytic activity groups. **(B)** Correlation analysis showing the relationship between levels of immune-infiltrating cells and glycolysis. **(C)** Comparative analysis of the proportions of 22 immune-infiltrating cell types within the high-immunity and low-immunity groups. *p<0.05, **p<0.01, ***p<0.001.

Prior research has suggested a significant link between glycolysis and tumor immunity, influencing the responsiveness of tumors to radiotherapy. To investigate this relationship, we employed the ssGSEA method to evaluate immune cell infiltration based on 29 established immune hallmark pathways. Our findings revealed that the group with low glycolytic activity displayed significant enrichment in various immune-related pathways (**Figure 2A**). Furthermore, to quantify the immune cell populations between the low-glycolysis and high-glycolysis groups, we utilized CIBERSORT analysis. Our correlation analyses uncovered a significant inverse relationship between the established radiosensitivity index and several immune cell types (**Figure 2B**). Notably, the low-glycolysis group exhibited higher levels of CD8+ T cells, follicular helper T cells, and regulatory T cells (Tregs). In contrast, the high-glycolysis group was characterized by an increased prevalence of M0 macrophages and resting natural killer (NK) cells (**Figure 2C**). These findings suggest a decline in the infiltration of T cells and macrophages in the high-glycolysis group, pointing to potential mechanisms of immune evasion.

### Single-cell RNA sequencing reveals the relationship between glycolytic activity and immune cell composition in HNSC

**Figure 3:**
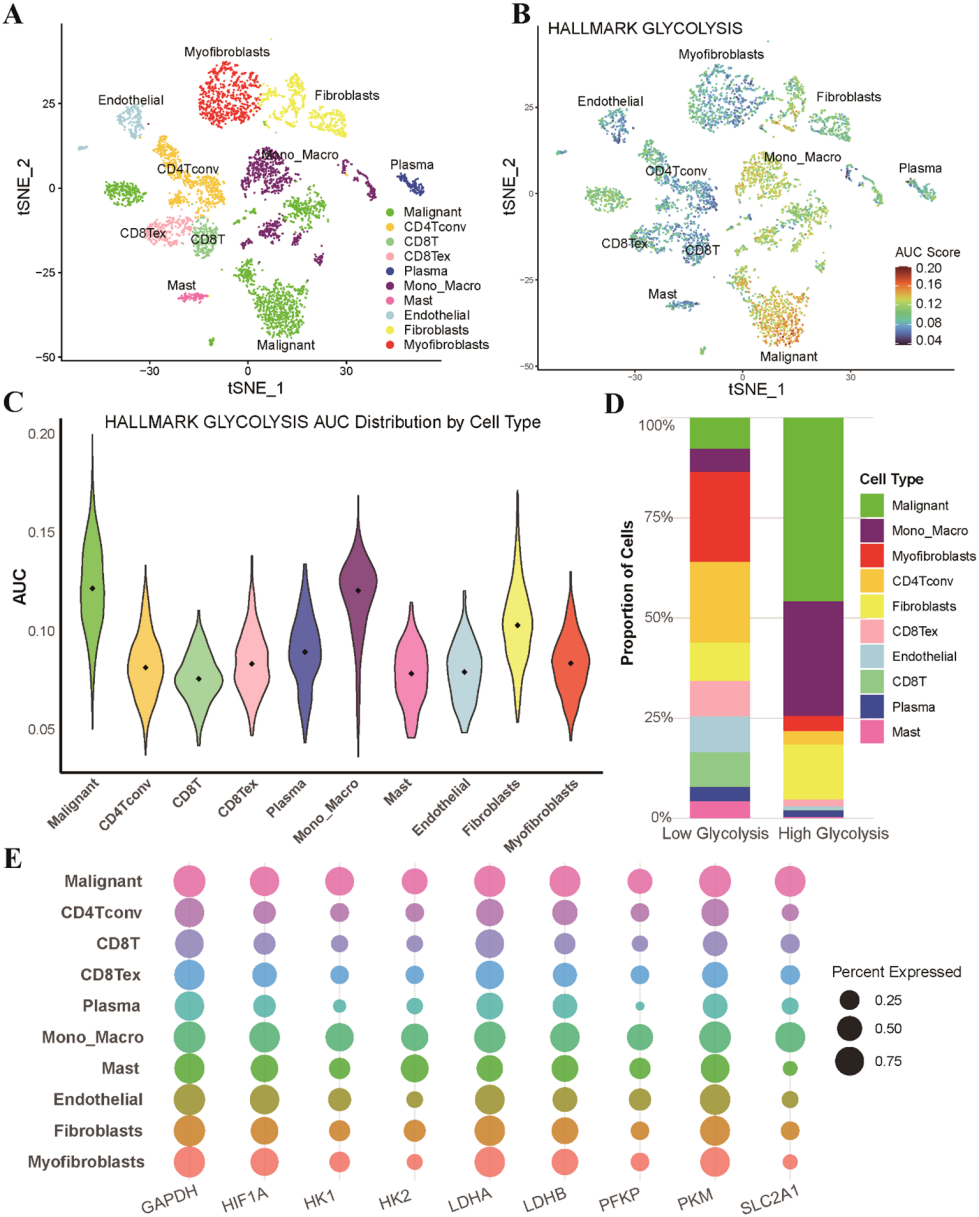
Evaluation of single-cell RNA sequencing characterization within the HNSC microenvironment. **(A)** t-SNE visualization highlighting primary cell types in the tumor immune microenvironment. **(B)** t-SNE representation of glycolytic levels across diverse cellular populations. **(C)** Violin plot showing the distribution of HALLMARK_GLYCOLYSIS AUCell scores across cell types. **(D)** Comparative analysis of cell type proportions in high versus low glycolysis score groups. **(E)** Variations in the expression of key glycolytic markers among different cell types.

To investigate the relationship between glycolytic activity and immune cell infiltration in HNSC, we analyzed single-cell RNA sequencing data (GSE103322) sourced from the TISCH2 platform. This dataset enabled us to categorize a total of 22 distinct cell types, identifying 11 specific subsets, including “Malignant cells, ” “Myofibroblasts, ” “Plasma cells, ” “Fibroblasts, ” “Myocytes, ” “Monocyte/Macrophages, ” “Endothelial cells, ” “CD4+ T conventional cells” (CD4+ Tconv), “Mast cells, ” “CD8+ T cells, ” and “Exhausted CD8+ T cells” (**Figure 3A**). Using the “AUCell” package, we calculated glycolytic scores for each of these subsets. The highest glycolysis scores were observed mainly in malignant cells and monocyte/macrophage populations, whereas conventional CD8+ T cells showed relatively lower scores. The Exhausted CD8+ T cells subset displayed only a modest increase compared with CD8+ T cells (**Figures 3B, C**). To further explore the impacts of glycolysis on immune cell composition, we classified the cells into low- and high-glycolysis subgroups based on the median glycolytic score. Despite an increased percentage of tumor cells in the high-glycolysis group, there was a noticeable decrease in the proportion of CD8+ T cells (**Figure 3D**). These findings align with the previous immunoassay results from the TCGA database. Additionally, we evaluated the expression levels of several glycolytic marker genes (including HK2, PKM, PFKP, HK1, GAPDH, SLC2A1, LDHA, LDHB, and HIF1A) across the identified cell subsets. The data revealed that tumor cells exhibited significantly higher expression levels of these markers compared to other cell types (**Figure 3E**). Collectively, these observations suggest that glycolytic processes are predominantly active within tumor cells, and that within this glycolytic environment, infiltration by macrophages, monocytes, and T cells is diminished, potentially indicating mechanisms of immune escape.

### Establishing a glycolysis-associated radiosensitivity model for HNSC patients

**Figure 4:**
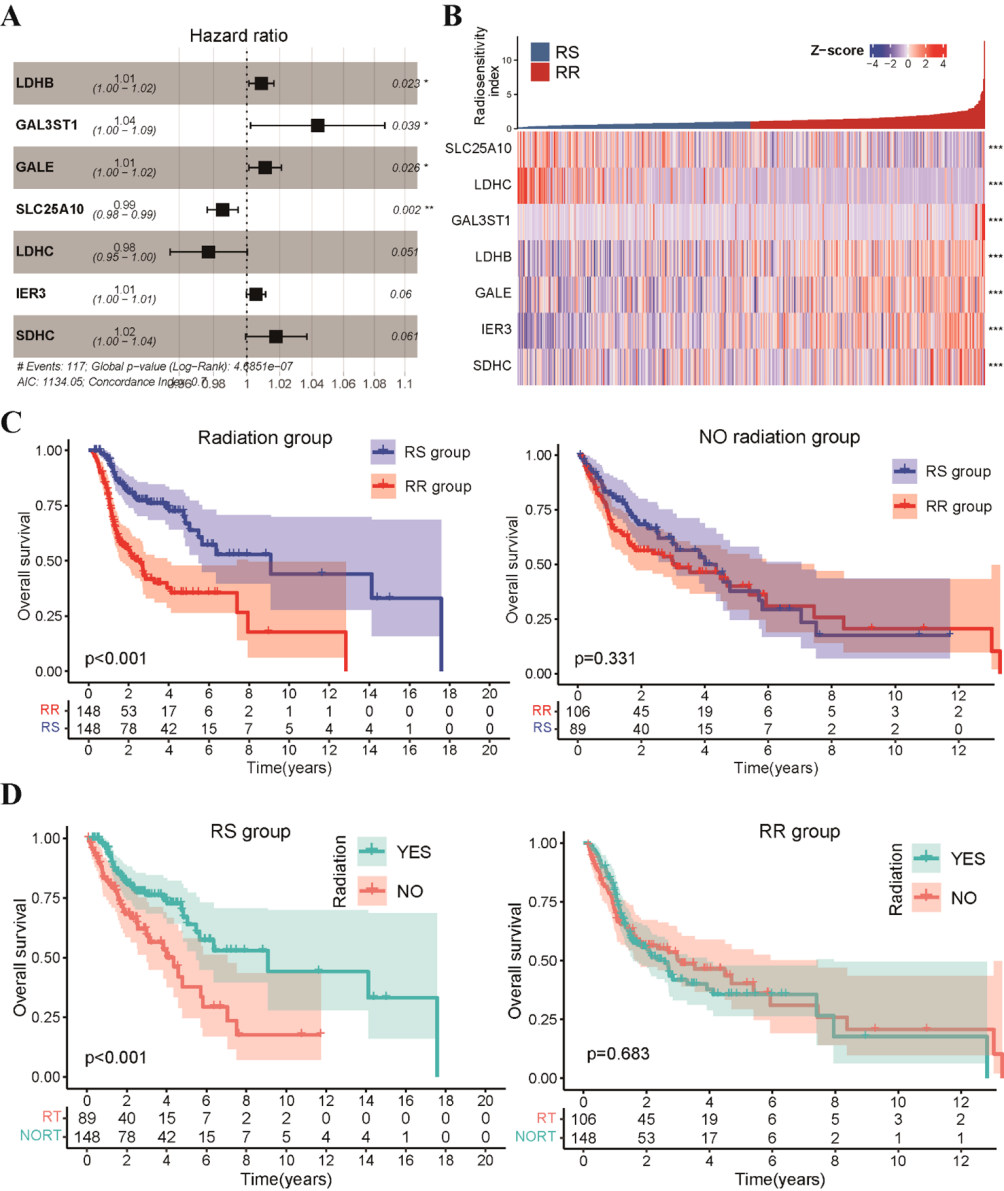
Establishing a glycolysis-associated radiosensitivity model in HNSC patients. **(A)** Forest plot displaying multivariate Cox regression results for the 7-gene index, including hazard ratios and 95% confidence intervals. **(B)** Heatmap illustrating the expression profiles of the seven RI component genes across RS and RR groups. **(C)** Overall survival curves comparing patients receiving and not receiving radiotherapy within both RS and RR groups. **(D)** Kaplan-Meier plots revealing OS differences based on radiosensitivity classification within RS and RR groups. *p<0.05, **p<0.01, ***p<0.001.


To assess the potential of glycolysis-related genes in predicting radiation response in HNSC, we constructed a radiosensitivity index (RI) using data from the TCGA cohort. We performed an initial univariate Cox regression analysis on 289 glycolysis-related genes to assess their individual association with OS in patients stratified by radiotherapy status (**Supplementary Figure 1A**). From this analysis, we revealed that a subset of 28 genes exhibited a significant correlation with OS exclusively in the radiotherapy cohort. In contrast, no such association was observed in patients who did not receive radiation therapy (**Supplementary Figure 1B**). Following this, we utilized multivariate Cox regression to establish a radiosensitivity index specifically for patients undergoing radiotherapy, resulting in a model composed of seven key genes (**Figure 4A**). The radiosensitivity index (RI) was calculated using the following formula:

$$RI=\;\left(expression\;of\;LDHB\;\times \;0.00874\right)\;+\;\left(expression\;of\;GAL3ST1\;\times \;0.04276\right)\;+\;\left(expression\;of\;GALE\;\times \;0.01110\right)\;+\;\left(expression\;of\;SLC25A10\;\times \;-0.01461\right)\;+\;\left(expression\;of\;LDHC\;\times \;-0.02317\right)\;+\;\left(expression\;of\;IER3\;\times \;0.00553\right)\;+\;\left(expression\;of\;SDHC\;\times \;0.01766\right)$$

Patients were then classified individuals into radiosensitive (RS) and radioresistant (RR) groups based on the median RI score across the entire HNSC cohort (**Figure 4B**). Kaplan-Meier survival analysis demonstrated a statistically significant improvement in OS for RS patients undergoing radiotherapy compared to those not receiving it. Conversely, in the RR group, radiotherapy conferred no significant OS advantage (**Figure 4C**). Further examination of the patients who underwent radiotherapy revealed that the RS group exhibited markedly prolonged OS relative to the RR group. No OS disparity was observed between RS and RR groups in the non-radiotherapy group (**Figure 4D**). Analysis of disease-specific survival (DSS) also demonstrated that the benefit of radiotherapy was largely confined to the RS patient subgroup (**Supplementary Figures 2A, B**). To evaluate the independent prognostic value of the model, we performed a multivariate Cox regression analysis including the RI, patient age, gender, tumor grade, TNM stage, radiation treatment, HPV, and *TP53* mutation status. The results demonstrated that the RI remained a significant independent prognostic factor for overall survival. This confirms its predictive capacity beyond serving merely as a surrogate for clinical stages or established molecular features such as HPV infection and TP53 mutations (**Supplementary Figure 2C**). These results indicate that this glycolysis-based RI shows promise as a predictive biomarker for radiotherapy outcomes in HNSC. The differential survival patterns observed suggest that the RI can effectively stratify patients who are likely to benefit from radiation therapy.

### Association of radiosensitivity model with clinical features and biological pathways

**Figure 5:**
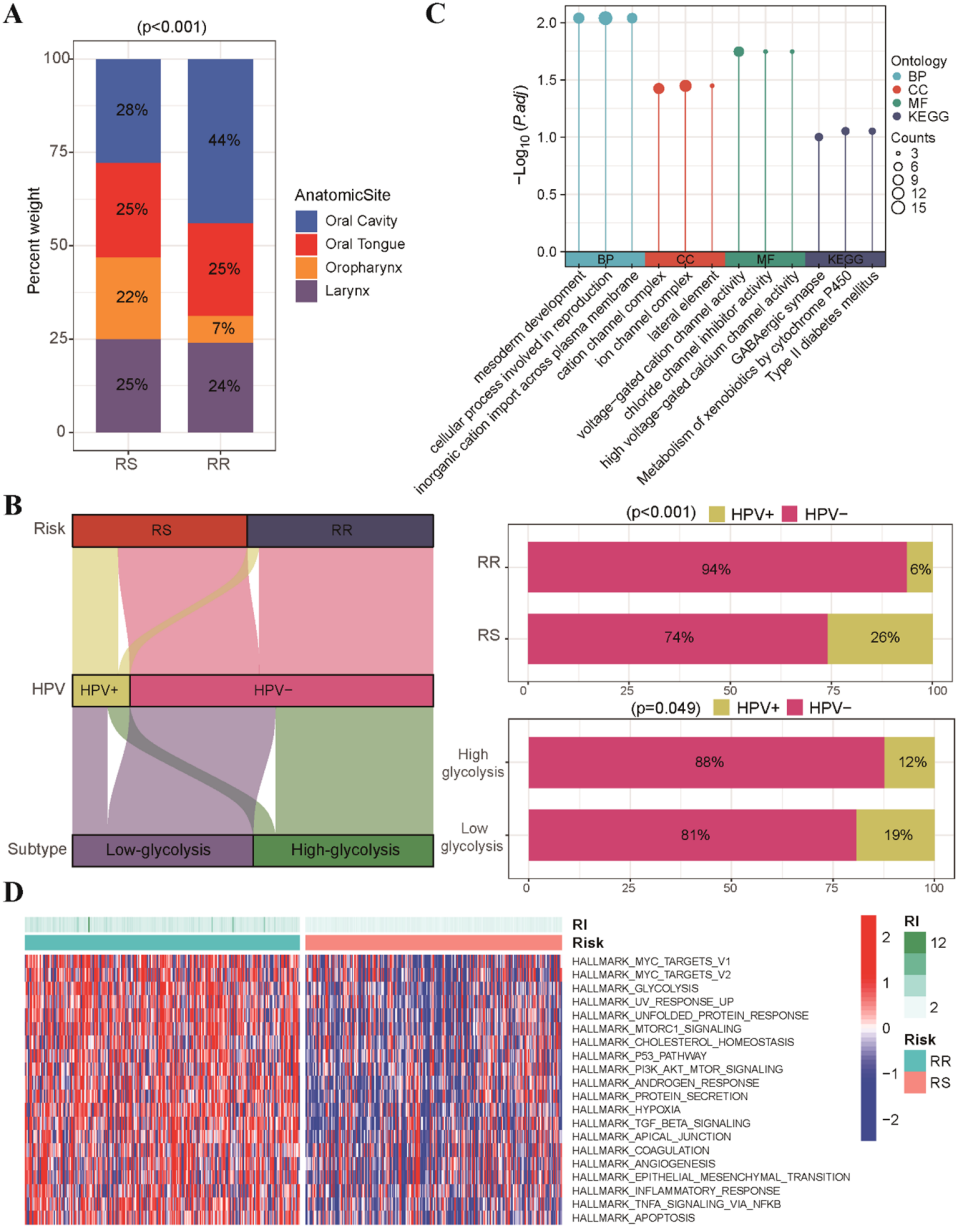
Molecular characterization of the radiosensitivity subgroup in HNSC. **(A)** tacked bar graph illustrating the distribution of patients across different anatomical sites between the RS and RR groups. **(B)** The Sankey diagram and stacked bar charts illustrate the distribution and significant enrichment of HPV-positive patients in the RS group and the low-glycolysis subtype. **(C)** Graph depicting the top enriched GO terms linked to DEGs, categorized into biological process (BP), cellular component (CC), and molecular function (MF). **(D)** Heatmap presenting enrichment scores for the most significantly differentially enriched hallmark pathways between two groups.

To investigate potential links between the developed radiosensitivity model and specific clinicopathological parameters, we compared patient characteristics between the RS and RR groups. Evaluation of T stage, N stage, M stage, pathological grade, age, and gender showed no statistically significant differences between the two groups (**Supplementary Figure 3A**). Interestingly, a notable trend emerged when examining the anatomical sites; the RS group displayed a significantly higher frequency of tumors originating in the oropharynx, while tumors located in the oral cavity were more frequently found in the RR group (**Figure 5A**). In line with this anatomical distribution, further stratification by HPV status revealed that HPV-positive tumors were significantly enriched in both the RS group and the low-glycolysis subtype compared to HPV-negative tumors. This strongly aligns with the established consensus that HPV-positive HNSC exhibits a less glycolytic and more radiosensitive phenotype (**Figure 5B**). Furthermore, given the critical role of TP53 in cellular metabolism and treatment response, we evaluated TP53 mutation profiles. We observed that TP53 mutations were significantly enriched in the RR group and were associated with higher RI (**Supplementary Figure 3B**), providing a strong mechanistic link between our radiosensitivity index and established genetic drivers of radiation resistance.

Further examination of gene expression profiles between the RS and RR groups identified a total of 215 differentially expressed genes (DEGs) exhibiting significant variance (**Supplementary Figure 3C**). Gene Ontology (GO) enrichment analysis demonstrated that these DEGs were primarily linked to processes such as inorganic cation transport across plasma membranes, ion channel complex involvement, voltage-gated cation channel activity, and GABAergic synapse functions. These findings suggest that these processes may play a critical role in determining radiosensitivity (**Figure 5C**). The GVSA demonstrated that the RR subgroup exhibited prominent enrichment of hallmark pathways involved in oncogenic signaling, including the PI3K/AKT/mTOR pathway, hypoxia-related responses, and angiogenic pathways (**Figure 5D**). Furthermore, we examined the connections between the expression levels of the seven genes in the radiosensitivity index and these key molecular pathways (**Supplementary Figure 4A**). To evaluate the broader metabolic context, we further evaluated the balance between glycolysis and oxidative phosphorylation. We observed that the Glycolysis-to-OXPHOS ratio was significantly higher in the RR group compared to the RS group and showed a positive correlation with the radiosensitivity index (**Supplementary Figure 4B**). Taken together, these results indicate that the radioresistant tumors identified by our model rely heavily on a metabolic transition from oxidative phosphorylation to glycolysis, supported by underlying hypoxia and PI3K/AKT/mTOR activation.

### Tumor immune microenvironment profiling in radiosensitivity model

**Figure 6:**
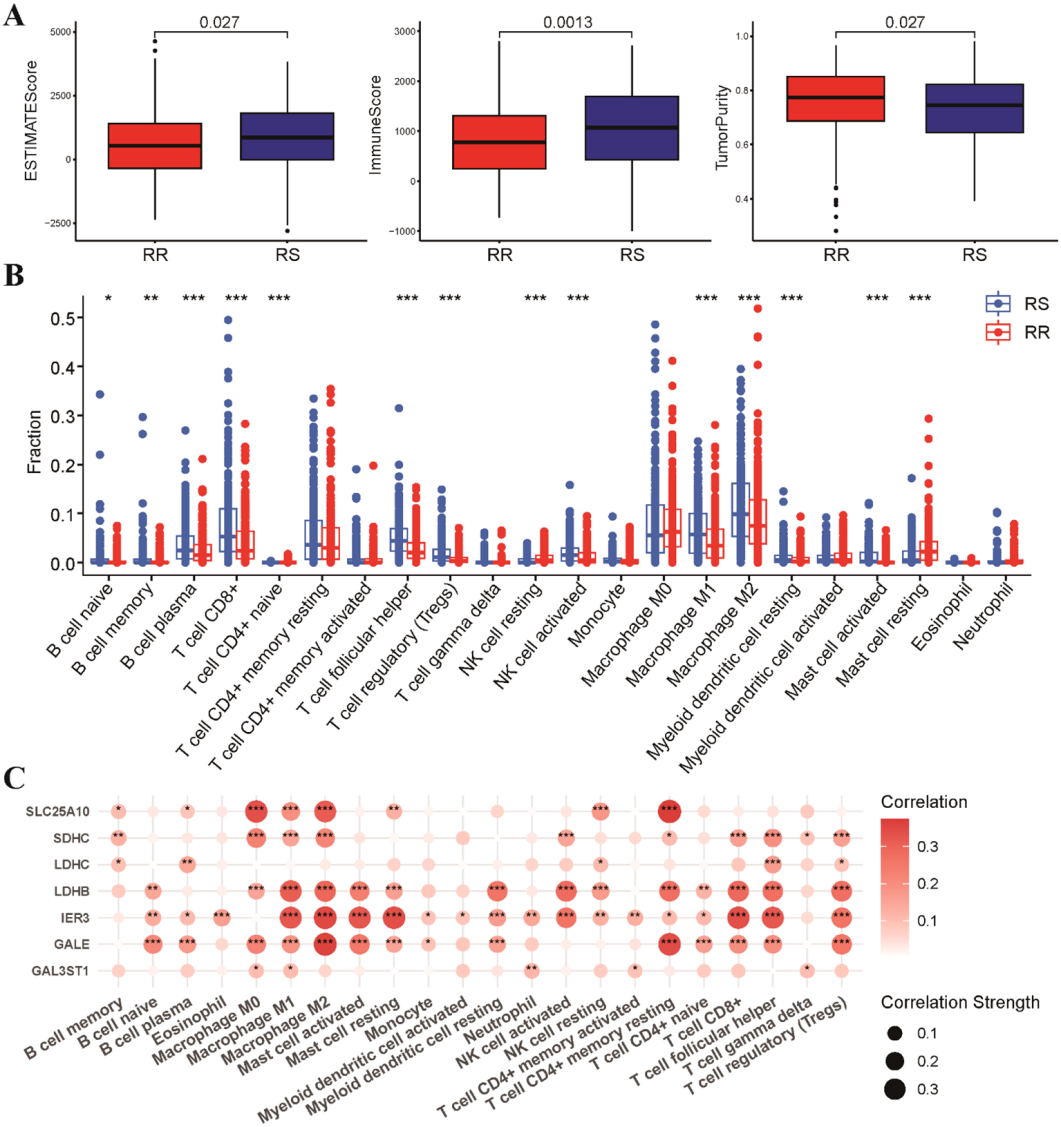
Comparative immune profiling in RS and RR groups. **(A)** Distribution of ESTIMATE scores, immune scores, and tumor purity in the RS and RR groups. **(B)** Immune cell composition profiles in the RS and RR groups, determined through CIBERSORT analysis. **(C)** Correlation analysis between the expression of key genes within the glycolysis-related radiosensitivity index and the abundance of various immune cell types. *p<0.05, **p<0.01, *** p<0.001.

Given the emerging significance of the tumor immune microenvironment (TIME) in modulating response to radiotherapy (29, 30), we sought to characterize the immune landscape associated with our radiosensitivity model. We began by analyzing the immune landscape of tumors using the ESTIMATE algorithm. The RS group displayed significantly elevated ESTIMATE and immune scores compared to the RR group. In contrast, the RR group displayed increased tumor purity, indicative of diminished immune and stromal cell infiltration (**Figure 6A**). To assess the differences in immune cells between these two groups, we employed CIBERSORT deconvolution analysis to quantify tumor-infiltrating lymphocyte populations. The results showed that the RS group had significantly elevated levels of CD8+ T cells, follicular helper T cells, and regulatory T cells. In contrast, the RR group was characterized by an increased proportion of resting mast cells and natural killer (NK) cells (**Figure 6B**). Furthermore, correlation analyses revealed an inverse relationship between the radiosensitivity index and these identified immune cell types (**Supplementary Figure 4C**). We also explored the associations between the expression levels of the three genes within our radiosensitivity index and the infiltration levels of immune cell types. Most immune cell types displayed positive correlations with the radiosensitivity index (**Figure 6C**). This is particularly significant, given role of CD8+ T cells in mediating antitumor immune responses, including triggered by radiotherapy. To validate these observations, we employed multiple independent algorithms, including TIMER, XCELL, MCPCOUNTER, and QUANTISEQ, to independently assess CD8+ T cell infiltration. All four algorithms consistently confirmed higher CD8 T cell abundance in the RS group compared to the RR group (**Supplementary Figure 4D**). These findings suggest that enhanced cytotoxic immune activity may contribute to the observed radiation responsiveness.

### Influence of the radiosensitivity model on therapeutic response

**Figure 7:**
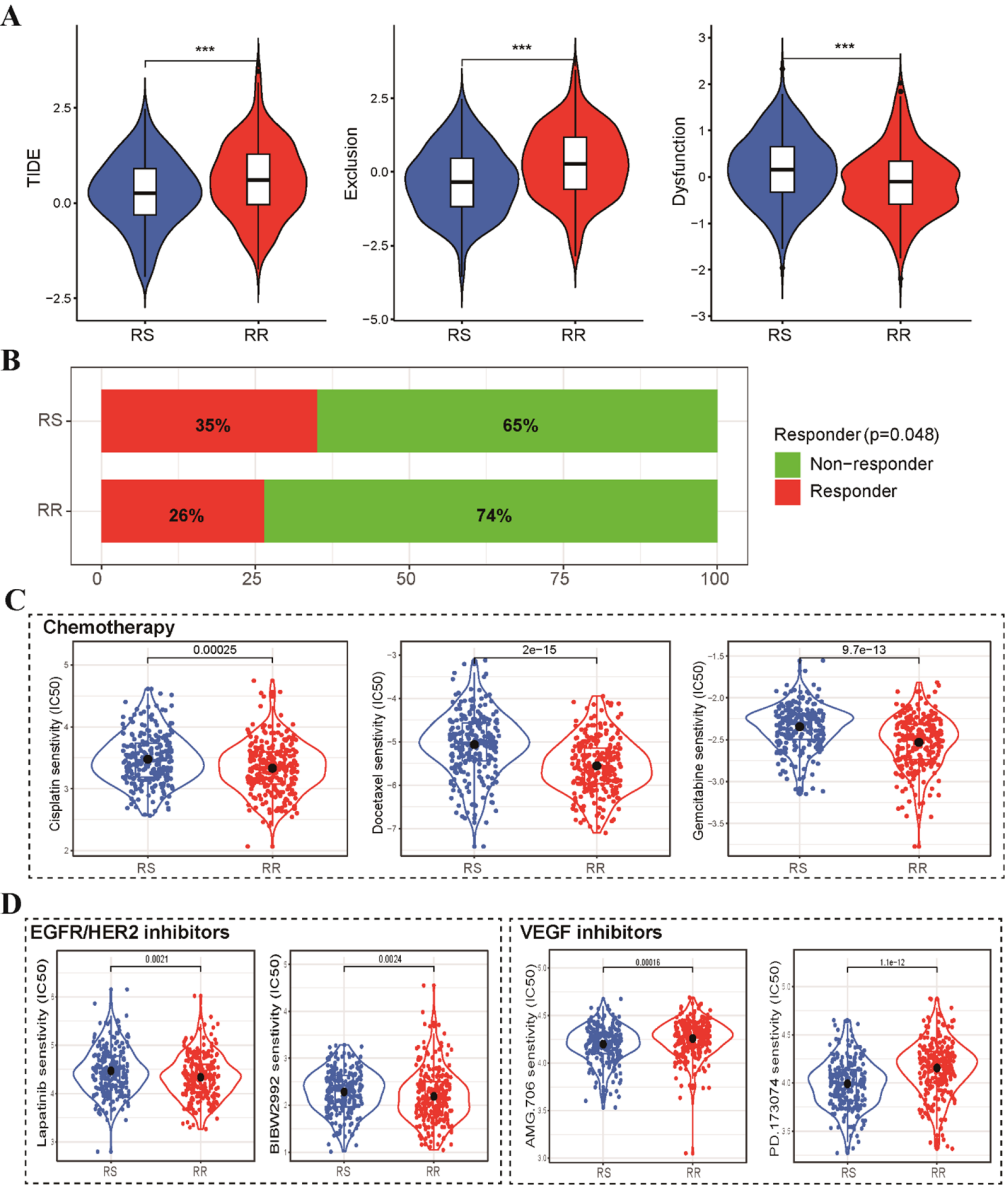
Therapeutic response heterogeneity between RS and RR groups. **(A)** Violin plots displaying the distribution of TIDE scores, exclusion scores, and dysfunction scores across the RS and RR groups. **(B)** Bar graph representing the proportion of patients categorized as responders or non-responders to immunotherapy within RS and RR groups. **(C)** Dot plots comparing IC50 values for cisplatin, docetaxel, and gemcitabine between RS and RR subgroups. **(D)** Dot plots illustrating differential IC50 values for EGFR/HER2 inhibitors and VEGF inhibitors in the two groups. ***p<0.001.

To assess the potential of the radiosensitivity index to inform therapeutic strategies, we examined its relationship with diverse cancer treatment approaches, encompassing immunotherapy, chemotherapy, and targeted therapies. Utilizing the TIDE algorithm, our analyses revealed that patients within the RS group displayed notably decreased TIDE scores and exclusion scores relative to the RR group, indicating a potentially enhanced response to immunotherapy (**Figure 7A**). There was a higher percentage of responders to immunotherapy in the RS group, suggesting a greater likelihood of positive therapeutic responses in these patients (**Figure 7B**). For chemotherapy sensitivity, we evaluated the estimated IC50 values of common chemotherapeutic drugs. Interestingly, patients in the RR group exhibited increased susceptibility to platinum, gemcitabine and docetaxel therapies, as indicated by lower IC50 thresholds (**Figure 7C**). Regarding targeted therapies, we noted distinct sensitivity patterns. Lower IC50 values for EGFR/HER2 inhibitors were observed in the RR group, indicating enhanced efficacy of these agents in radioresistant tumors. Conversely, the RS group exhibited greater sensitivity to VEGF inhibitors (**Figure 7D**). Taken together, these data emphasize the promise of incorporating radiosensitivity index into treatment decision-making, with the goal of improving individualized treatment approaches in HNSC.

### Validation of radiosensitivity index and glycolysis in HNSC cells

**Figure 8:**
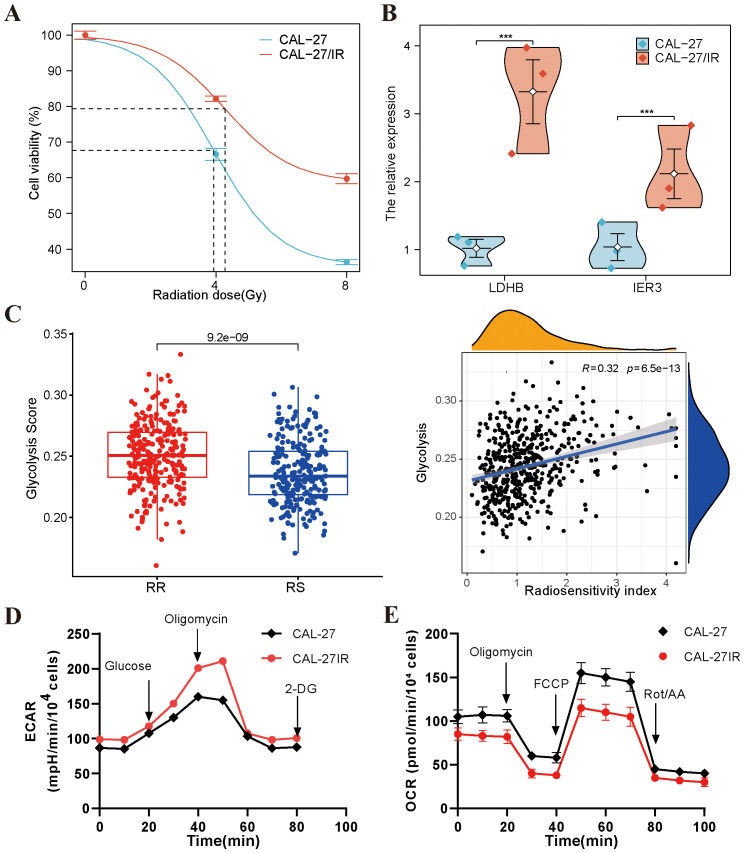
Experimental validation of radiosensitivity and glycolytic activity in HNSC Cell. **(A)** Dose-dependent cell viability curves of CAL-27 and CAL-27IR cells following ionizing radiation **(B)** Relative mRNA expression levels of IER3 and LDHB in CAL-27 and CAL-27IR cells. **(C)** Scatter plot of glycolysis scores in RS and RR subgroups from TCGA-HNSC, with Pearson correlation analysis between glycolysis scores and radiosensitivity index. **(D)** ECAR profiles illustrating glycolytic flux in CAL-27 and CAL-27IR cells following sequential exposure to glucose, oligomycin, and 2-deoxyglucose. **(E)** OCR profiles of CAL-27 and CAL-27IR cells assessed over time with sequential addition of oligomycin, FCCP, and Rot/AA. Data are normalized to cell number. ***p<0.001.

To experimentally validate the radiosensitivity index identified through computational analysis, we employed a non-radioresistant TSCC cell line CAL-27 and its radioresistant counterpart, CAL-27IR. Cell viability assays indicated that CAL-27IR cells exhibited significantly higher viability at all tested doses compared to CAL-27, confirming their radioresistant phenotype (**Figure 8A**). For subsequent RT-PCR validation, we specifically selected LDHB and IER3 among the modeled genes. LDHB is directly involved in the core glycolytic pathway, and IER3 is highly responsive to cellular stress, making them highly relevant to our study’s metabolic theme. Quantitative RT-PCR analysis revealed that IER3 and LDHB, two key genes identified in our radiosensitivity index, were markedly upregulated in CAL-27IR cells relative to the parental line (**Figure 8B**). Given the critical role of glycolysis in tumor energy metabolism and its association with radio-resistance, we examined glycolysis scores in the RR and RS groups using ssGSEA analysis of the TCGA-HNSC dataset. The results confirmed a significant elevation of glycolysis scores in the RR group relative to the RS group. A strong positive correlation between glycolysis scores and radiosensitivity index was observed (**Figure 8C**). Furthermore, ECAR measurements revealed an increased glycolytic rate in CAL-27IR cells compared to CAL-27 cells, indicative of heightened glycolytic activity (**Figure 8D**). Concurrently, CAL-27IR cells displayed a reduced oxygen consumption rate (OCR) compared to CAL-27 cells (**Figure 8E**). These findings offer experimental support for a mechanistic link between altered expression of key radiosensitivity genes and increased glycolysis contribute to radiation resistance, potentially representing a targetable vulnerability in HNSC.

## Discussion

The present study reveals a critical interplay between glycolytic activity and radiosensitivity in HNSC. We found that the level of glycolytic activity significantly influences how HNSC tumors respond to radiation. Tumors with low glycolytic activity exhibit a greater survival benefit from radiotherapy compared to those with high glycolytic activity. This finding highlights the importance of glycolysis as a key factor in determining radiation sensitivity in HNSC. This observation is in line with other studies indicating that higher glucose metabolism is often associated with resistance to radiation therapy (31, 32). In our study, we used two related approaches to evaluate glycolysis. First, we applied a standard 289-gene set to calculate an overall glycolysis score for each patient. While a large number of genes is necessary to accurately assess general pathway activity, testing hundreds of genes is difficult in routine clinical practice. Therefore, we filtered this initial group down to seven key genes using sequential regression analysis to create the RI model. Importantly, these seven genes are a direct subset of the original 289-gene pool. The strong positive correlation between the RI and the overall glycolysis score confirms that our 7-gene model still accurately reflects the patient’s general metabolic state. This approach allowed us to develop a practical prognostic tool while maintaining the same biological meaning throughout the evaluation.

The differential response to radiotherapy based on glycolytic activity appears to be closely linked to the TIME (33). High glycolytic activity has been previously associated with aggressive tumor phenotypes and an enhanced capacity for immune evasion (34). Our findings indicate that low-glycolysis tumors are characterized by a more robust infiltration of immune cells, particularly CD8+ T cells, follicular helper T cells, and regulatory T cells. This contrasts with high-glycolysis tumors, which exhibit a relative paucity of T cell infiltration and a higher prevalence of M0 macrophages and resting natural killer (NK) cells. The significant inverse relationship between glycolytic activity and specific immune cell populations suggests that tumors exhibiting high glycolysis may employ metabolic reprogramming to create an immunosuppressive microenvironment, thereby undermining effector T cell functions and promoting their own survival (35). Using single-cell RNA sequencing data, we further revealed that malignant cells and macrophages exhibit elevated glycolytic activity within the HNSC microenvironment. Importantly, the high-glycolysis subgroup demonstrated a decreased proportion of CD8+ T cells, strengthening the idea that increased glycolysis is associated with reduced cytotoxic T cell infiltration. The elevated expression of glycolytic marker genes in tumor cells further supports the hypothesis that tumor cell metabolism plays a central role in shaping the immune landscape and influencing radiotherapy outcomes (34).

To convert these findings into a clinically applicable tool, we developed a glycolysis-associated RI based on the expression of seven key genes. This RI effectively stratified HNSC patients into RS and RR groups. Kaplan-Meier survival analysis demonstrated that radiotherapy significantly improved OS in RS patients but conferred no such benefit in RR patients, highlighting the potential of the RI to predict which patients are most likely to benefit from radiation therapy. Interestingly, the RI also correlated with anatomical location, with oropharyngeal tumors appearing more frequently in the RS group, while oral cavity tumors were more prevalent in the RR group. This finding suggests further investigation to determine if site-specific metabolic differences contribute to the observed differences in radiosensitivity (36). Furthermore, GSEA revealed that the RR subgroup exhibited enrichment of oncogenic signaling pathways, including PI3K/AKT/mTOR, hypoxia-related responses, and angiogenesis (37). This suggests that these pathways may contribute to radio-resistance by promoting tumor cell survival, proliferation, and angiogenesis in the face of radiation-induced stress.

Finally, we explored the potential of the RI to inform therapeutic strategies beyond radiotherapy. Our findings highlight the potential of the RI to guide personalized treatment strategies in HNSC, particularly by leveraging the interplay between radiotherapy, immune infiltration, and targeted therapies. The enhanced CD8 T cell infiltration observed in RS tumors, coupled with lower TIDE scores indicative of improved immunotherapy responsiveness, suggests that these patients may derive significant benefit from combining radiotherapy with immune checkpoint inhibitors to further augment the cytotoxic anti-tumor response (38). Conversely, the cold immune microenvironment of RR tumors, characterized by a relative lack of T cell infiltration, suggests that these patients may be less likely to respond to immunotherapy alone and may instead benefit from strategies that combine radiotherapy with chemotherapy or targeted therapies, such as EGFR/HER2 inhibitors (39). These findings emphasize the potential of integrating the RI into clinical practice to guide personalized treatment approaches, with the ultimate goal of optimizing therapeutic responses by matching treatment strategies with the immune landscape of each patient’s tumor.

Our study has some limitations that should be acknowledged. First, the retrospective nature of the TCGA data limits our ability to draw definitive conclusions about causality. Second, the RI was developed and internally assessed within a single TCGA-HNSC cohort, and no suitable independent public HNSC dataset with matched transcriptomic data, radiotherapy annotation, and survival follow-up was available for external validation. Third, while our *in vitro* experiments provide support for a link between glycolysis and radio-resistance, additional *in vivo* studies are needed to fully elucidate the underlying mechanisms. Finally, the clinical data did not allow us to correlate specific glycolysis inhibitors with the response to radiation. Future research should focus on investigating the mechanisms by which glycolysis modulates the TIME and influences immune cell infiltration. Furthermore, as our *in vitro* validation relied on monoculture CAL-27IR cells, the immune exclusion phenotype was inferred solely from bioinformatic analyses and requires further validation in tumor-immune models.

## Conclusion

In summary, our study reveals a significant relationship between glycolytic activity, immune cell infiltration, and radiosensitivity in HNSC. The glycolysis-associated radiosensitivity index we developed shows promise as a predictive biomarker for radiotherapy outcomes and could potentially be used to personalize treatment approaches in HNSC. Future research should focus on validating these findings in prospective clinical trials and exploring the potential of targeting glycolysis to improve radiotherapy efficacy.

## Data Availability

The datasets presented in this study can be found in online repositories. The names of the repository/repositories and accession number(s) can be found in the article/**Supplementary Material**.

## References

[1] Filho AM Laversanne M Ferlay J Colombet M Piñeros M Znaor A . The GLOBOCAN 2022 cancer estimates: Data sources, methods, and a snapshot of the cancer burden worldwide. Int J Cancer. (2025) 156:1336–46. doi: 10.1002/ijc.35278 39688499

[2] Tang E Lahmi L Meillan N Pietta G Albert S Maingon P . Treatment strategy for distant synchronous metastatic head and neck squamous cell carcinoma. Curr Oncol Rep. (2019) 21:102. doi: 10.1007/s11912-019-0856-5 31728650

[3] Hutchinson MND Mierzwa M D'Silva NJ . Radiation resistance in head and neck squamous cell carcinoma: Dire need for an appropriate sensitizer. Oncogene. (2020) 39:3638–49. doi: 10.1038/s41388-020-1250-3 32157215 PMC7190570

[4] Barba I Carrillo-Bosch L Seoane J . Targeting the Warburg effect in cancer: Where do we stand? Int J Mol Sci. (2024) 25:1–17. doi: 10.3390/ijms25063142 38542116 PMC10970388

[5] Paul S Ghosh S Kumar S . Tumor glycolysis, an essential sweet tooth of tumor cells. Semin Cancer Biol. (2022) 86:1216–30. doi: 10.1016/j.semcancer.2022.09.007 36330953

[6] Xiao J Li W Tan G Gao R . The gene signature linked to lactate metabolism predicts the prognosis and correlates with the immune status of head and neck squamous cell carcinoma. Front Genet. (2025) 16:1540841. doi: 10.3389/fgene.2025.1540841 40255484 PMC12006151

[7] Liu LX Heng JH Deng DX Zhao H Zheng ZY Liao LD . Sulconazole induces PANoptosis by triggering oxidative stress and inhibiting glycolysis to increase radiosensitivity in esophageal cancer. Mol Cell Proteomics MCP. (2023) 22:100551. doi: 10.1016/j.mcpro.2023.100551 37076047 PMC10205543

[8] Leimgruber A Hickson K Lee ST Gan HK Cher LM Sachinidis JI . Spatial and quantitative mapping of glycolysis and hypoxia in glioblastoma as a predictor of radiotherapy response and sites of relapse. Eur J Nucl Med Mol Imaging. (2020) 47:1476–85. doi: 10.1007/s00259-020-04706-0 32025750

[9] Chen F Tang C Yang F Ekpenyong A Qin R Xie J . HSP90 inhibition suppresses tumor glycolytic flux to potentiate the therapeutic efficacy of radiotherapy for head and neck cancer. Sci Adv. (2024) 10:eadk3663. doi: 10.1126/sciadv.adk3663 38394204 PMC10889358

[10] Hong M Tao S Zhang L Diao LT Huang X Huang S . RNA sequencing: New technologies and applications in cancer research. J Hematol Oncol. (2020) 13:166. doi: 10.1186/s13045-020-01005-x 33276803 PMC7716291

[11] Xiao Q Zhang F Xu L Yue L Kon OL Zhu Y . High-throughput proteomics and AI for cancer biomarker discovery. Adv Drug Delivery Rev. (2021) 176:113844. doi: 10.1016/j.addr.2021.113844 34182017

[12] Forker LJ Choudhury A Kiltie AE . Biomarkers of tumour radiosensitivity and predicting benefit from radiotherapy. Clin Oncol (Royal Coll Radiologists (Great Britain)). (2015) 27:561–9. doi: 10.1016/j.clon.2015.06.002 26119726

[13] Eschrich S Zhang H Zhao H Boulware D Lee JH Bloom G . Systems biology modeling of the radiation sensitivity network: A biomarker discovery platform. Int J Radiat Oncol Biol Phys. (2009) 75:497–505. doi: 10.1016/j.ijrobp.2009.05.056 19735874 PMC2762403

[14] Kim HS Kim SC Kim SJ Park CH Jeung HC Kim YB . Identification of a radiosensitivity signature using integrative metaanalysis of published microarray data for NCI-60 cancer cells. BMC Genomics. (2012) 13:348. doi: 10.1186/1471-2164-13-348 22846430 PMC3472294

[15] Wang S Xiong Y Zhao L Gu K Li Y Zhao F . UCSCXenaShiny: An R/CRAN package for interactive analysis of UCSC Xena data. Bioinf (Oxford England). (2022) 38:527–9. doi: 10.1093/bioinformatics/btab561 34323947 PMC8723150

[16] Liberzon A Birger C Thorvaldsdóttir H Ghandi M Mesirov JP Tamayo P . The Molecular Signatures Database (MSigDB) hallmark gene set collection. Cell Syst. (2015) 1:417–25. doi: 10.1016/j.cels.2015.12.004 26771021 PMC4707969

[17] Hänzelmann S Castelo R Guinney J . GSVA: Gene set variation analysis for microarray and RNA-seq data. BMC Bioinf. (2013) 14:7. doi: 10.1186/1471-2105-14-7 23323831 PMC3618321

[18] Sun D Wang J Han Y Dong X Ge J Zheng R . TISCH: A comprehensive web resource enabling interactive single-cell transcriptome visualization of tumor microenvironment. Nucleic Acids Res. (2021) 49:D1420–30. doi: 10.1093/nar/gkaa1020 33179754 PMC7778907

[19] Puram SV Tirosh I Parikh AS Patel AP Yizhak K Gillespie S . Single-cell transcriptomic analysis of primary and metastatic tumor ecosystems in head and neck cancer. Cell. (2017) 171:1611–1624.e24. doi: 10.1016/j.cell.2017.10.044 29198524 PMC5878932

[20] Butler A Hoffman P Smibert P Papalexi E Satija R . Integrating single-cell transcriptomic data across different conditions, technologies, and species. Nat Biotechnol. (2018) 36:411–20. doi: 10.1038/nbt.4096 29608179 PMC6700744

[21] Hu C Li T Xu Y Zhang X Li F Bai J . CellMarker 2.0: An updated database of manually curated cell markers in human/mouse and web tools based on scRNA-seq data. Nucleic Acids Res. (2023) 51:D870–6. doi: 10.1093/nar/gkac947 36300619 PMC9825416

[22] Aibar S González-Blas CB Moerman T Huynh-Thu VA Imrichova H Hulselmans G . SCENIC: Single-cell regulatory network inference and clustering. Nat Methods. (2017) 14:1083–6. doi: 10.1038/nmeth.4463 28991892 PMC5937676

[23] Bindea G Mlecnik B Tosolini M Kirilovsky A Waldner M Obenauf AC . Spatiotemporal dynamics of intratumoral immune cells reveal the immune landscape in human cancer. Immunity. (2013) 39:782–95. doi: 10.1016/j.immuni.2013.10.003 24138885

[24] Yoshihara K Shahmoradgoli M Martínez E Vegesna R Kim H Torres-Garcia W . Inferring tumour purity and stromal and immune cell admixture from expression data. Nat Commun. (2013) 4:2612. doi: 10.1038/ncomms3612 24113773 PMC3826632

[25] Chen B Khodadoust MS Liu CL Newman AM Alizadeh AA . Profiling tumor infiltrating immune cells with CIBERSORT. Methods Mol Biol. (2018) 1711:243–59. doi: 10.1007/978-1-4939-7493-1_12 29344893 PMC5895181

[26] Jiang P Gu S Pan D Fu J Sahu A Hu X . Signatures of T cell dysfunction and exclusion predict cancer immunotherapy response. Nat Med. (2018) 24:1550–8. doi: 10.1038/s41591-018-0136-1 30127393 PMC6487502

[27] Geeleher P Cox N Huang R . pRRophetic: An R package for prediction of clinical chemotherapeutic response from tumor gene expression levels. PloS One. (2014) 9:e107468. doi: 10.1371/journal.pone.0107468 25229481 PMC4167990

[28] Zhou ZR Wang XY Yu XL Mei X Chen XX Hu QC . Building radiation-resistant model in triple-negative breast cancer to screen radioresistance-related molecular markers. Ann Transl Med. (2020) 8:108. doi: 10.21037/atm.2019.12.114 32175401 PMC7049038

[29] Charpentier M Spada S Van Nest SJ Demaria S . Radiation therapy-induced remodeling of the tumor immune microenvironment. Semin Cancer Biol. (2022) 86:737–47. doi: 10.1016/j.semcancer.2022.04.003 35405340

[30] Guo S Yao Y Tang Y Xin Z Wu D Ni C . Radiation-induced tumor immune microenvironments and potential targets for combination therapy. Signal Transduction Targeted Ther. (2023) 8:205. doi: 10.1038/s41392-023-01462-z 37208386 PMC10199044

[31] Fang Y Zhan Y Xie Y Du S Chen Y Zeng Z . Integration of glucose and cardiolipin anabolism confers radiation resistance of HCC. Hepatol (Baltimore Md). (2022) 75:1386–401. doi: 10.1002/hep.32177 34580888 PMC9299851

[32] Xiao C Hou G Wang C Huang Y Liu Z . METTL1 mediates m7G modification of PFKFB3 mRNA to promote radioresistance in esophageal cancer by affecting glycolytic metabolism. Pathol Res Pract. (2025) 272:156102. doi: 10.1016/j.prp.2025.156102 40633178

[33] Liu J Cao X . Glucose metabolism of TAMs in tumor chemoresistance and metastasis. Trends Cell Biol. (2023) 33:967–78. doi: 10.1016/j.tcb.2023.03.008 37080816

[34] Wu L Jin Y Zhao X Tang K Zhao Y Tong L . Tumor aerobic glycolysis confers immune evasion through modulating sensitivity to T cell-mediated bystander killing via TNF-α. Cell Metab. (2023) 35:1580–1596.e9. doi: 10.1016/j.cmet.2023.07.001 37506695

[35] Jiang Z Liu Z Li M Chen C Wang X . Increased glycolysis correlates with elevated immune activity in tumor immune microenvironment. EBioMedicine. (2019) 42:431–42. doi: 10.1016/j.ebiom.2019.03.068 30935888 PMC6491961

[36] Ohashi T Terazawa K Shibata H Inoue N Ogawa T . Metabolic profiling analysis of head and neck squamous cell carcinoma. Oral Dis. (2024) 30:342–52. doi: 10.1111/odi.14432 36349421

[37] Aguayo F Perez-Dominguez F Osorio JC Oliva C Calaf GM . PI3K/AKT/mTOR signaling pathway in HPV-driven head and neck carcinogenesis: Therapeutic implications. Biology. (2023) 12 (5):672. doi: 10.3390/biology12050672 37237486 PMC10215516

[38] Galluzzi L Humeau J Buqué A Zitvogel L Kroemer G . Immunostimulation with chemotherapy in the era of immune checkpoint inhibitors. Nat Rev Clin Oncol. (2020) 17:725–41. doi: 10.1038/s41571-020-0413-z 32760014

[39] Sola AM Johnson DE Grandis JR . Investigational multitargeted kinase inhibitors in development for head and neck neoplasms. Expert Opin Invest Drugs. (2019) 28:351–63. doi: 10.1080/13543784.2019.1581172 30753792 PMC6857634

