# Supplementary material for: Evaluating glycolysis-associated biomarkers for radiotherapy sensitivity in head and neck squamous cancer
Source: Front Immunol. 2026 Jun 29;17:1736778. doi: 10.3389/fimmu.2026.1736778 (PMC13357613; doi:10.3389/fimmu.2026.1736778)
Supplement: Supplementary file 1 [file DataSheet1.pdf]

Supplement Figures

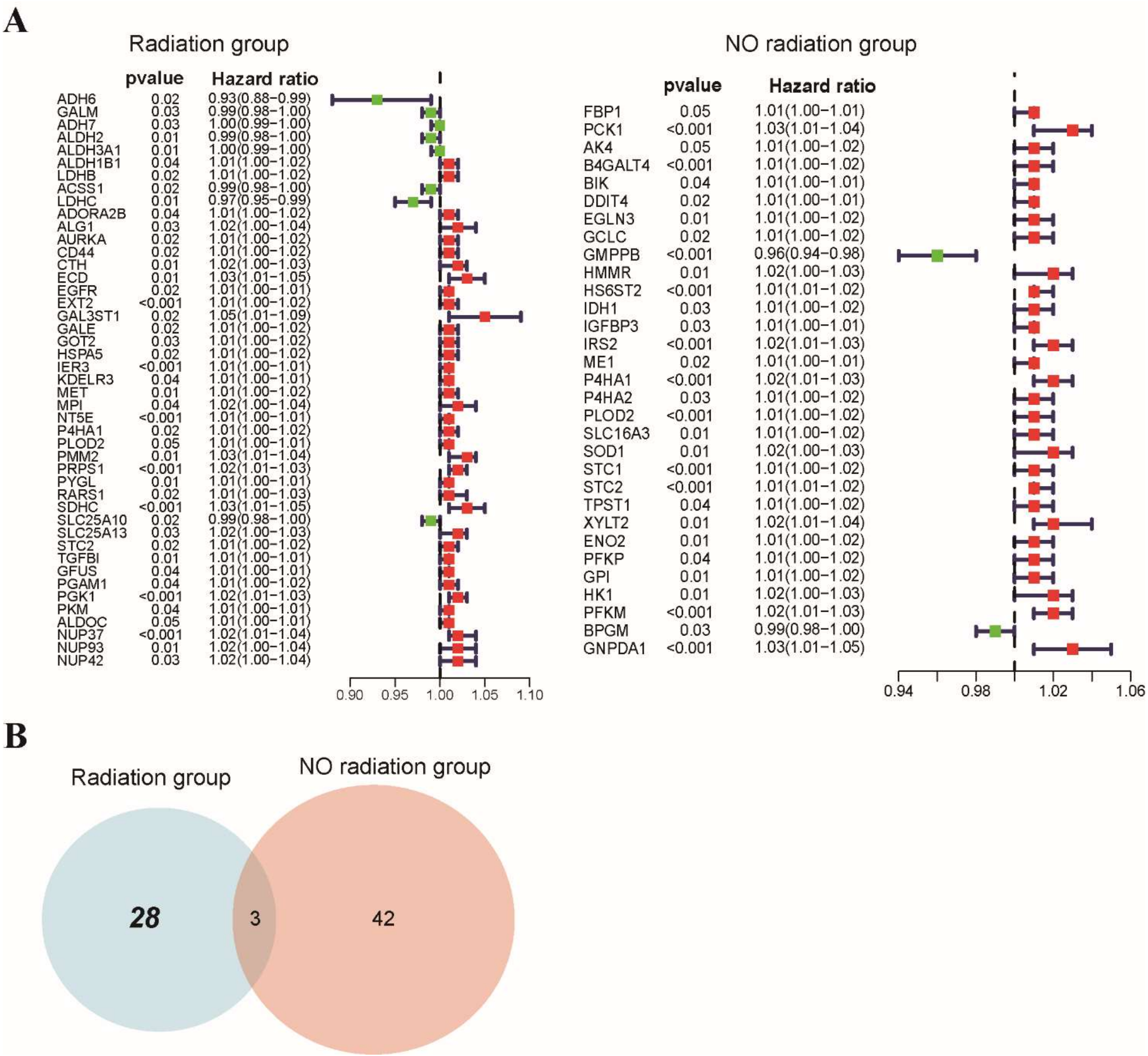

Figure S1 (A) Forest plot demonstrating the results from a univariate Cox proportional hazards analysis comparing outcomes in patients undergoing radiotherapy versus those who did not receive radiotherapy. **(B)** Venn diagram depicting the intersection of glycolysis-associated genes that are significantly correlated with overall survival (OS), revealing that 28 genes were identified as significant within the radiotherapy group but not in the group without radiotherapy.

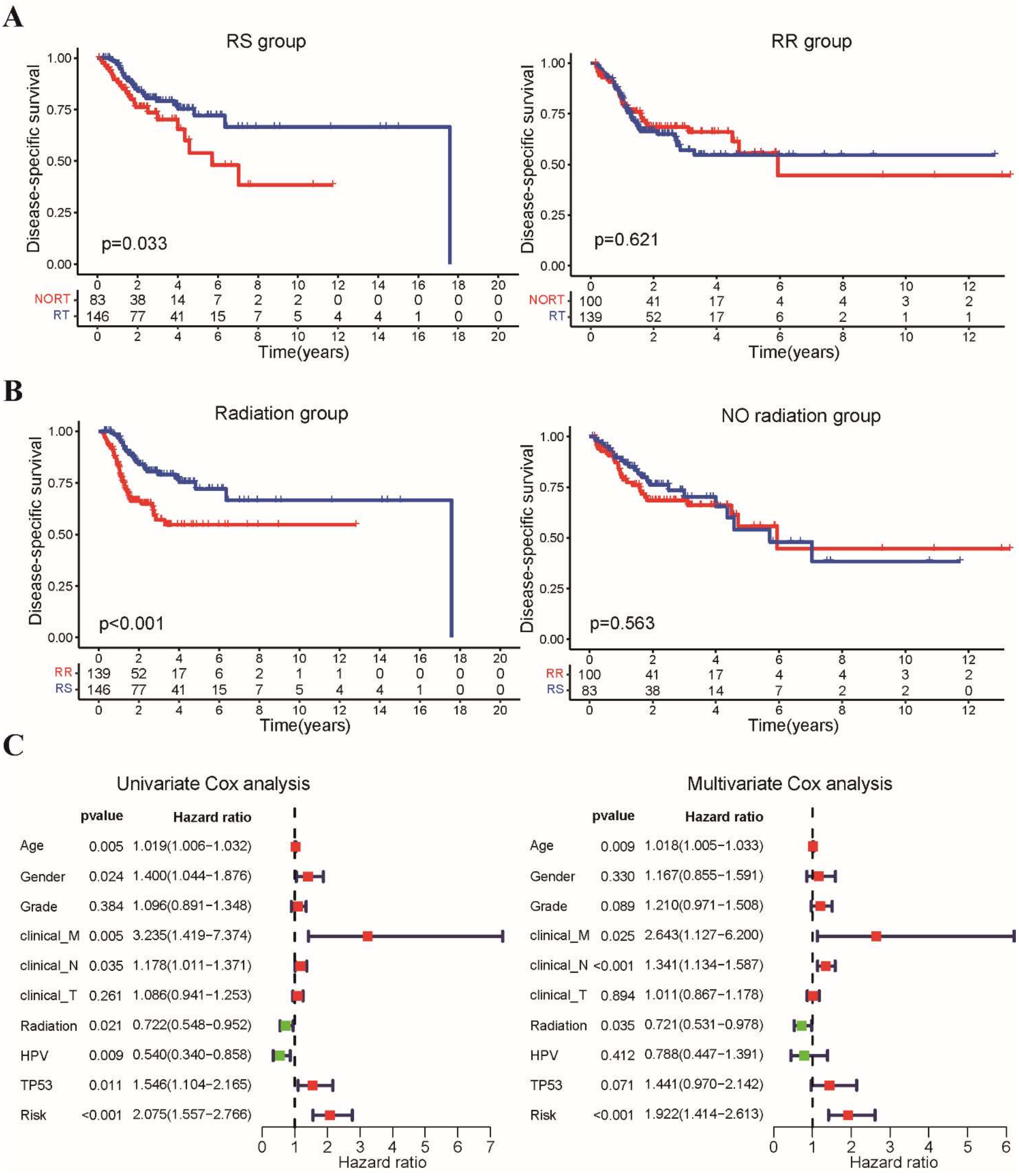

Figure S2 (A) Kaplan-Meier survival analysis highlighting differences in disease-specific survival (DSS) among radiotherapy patients classified into RS and RR categories. **(B)** Kaplan-Meier survival curves illustrating DSS within the TCGA cohort, stratified by radiosensitivity categorization (RS versus RR) and radiotherapy status. **(C)** Univariate and multivariate Cox regression analyses demonstrate that the RI serves as an independent prognostic factor adjusting for key clinical and molecular covariates.

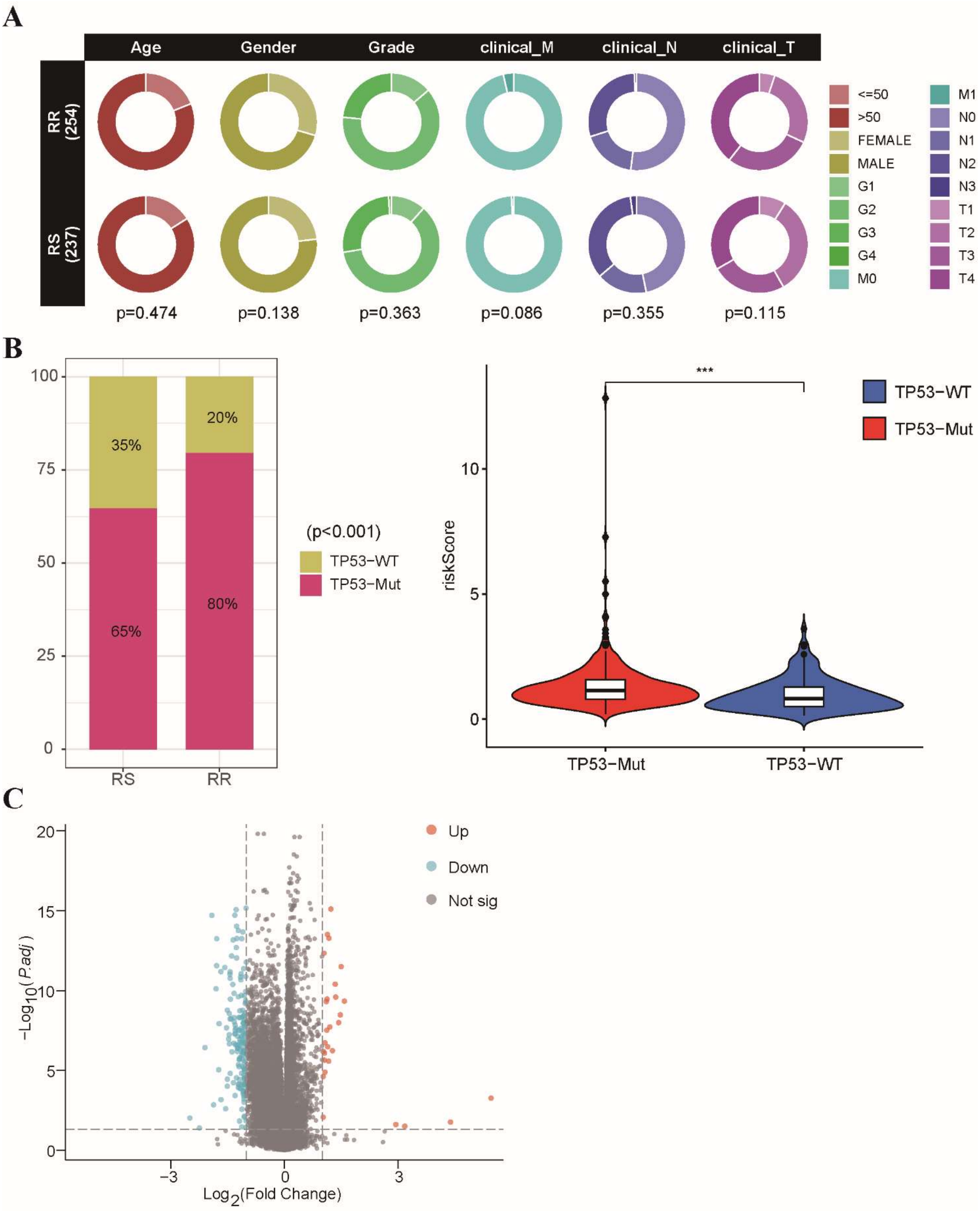

Figure S3 (A) Summary highlighting the relationship between glycolytic activity and clinical-pathological features in patients diagnosed with HNSC. **(B)** Association of TP53 mutation status with radiosensitivity (RS/RR) and risk scores. **(C)** Volcano plot exhibiting differentially expressed genes between the RS and RR classifications.

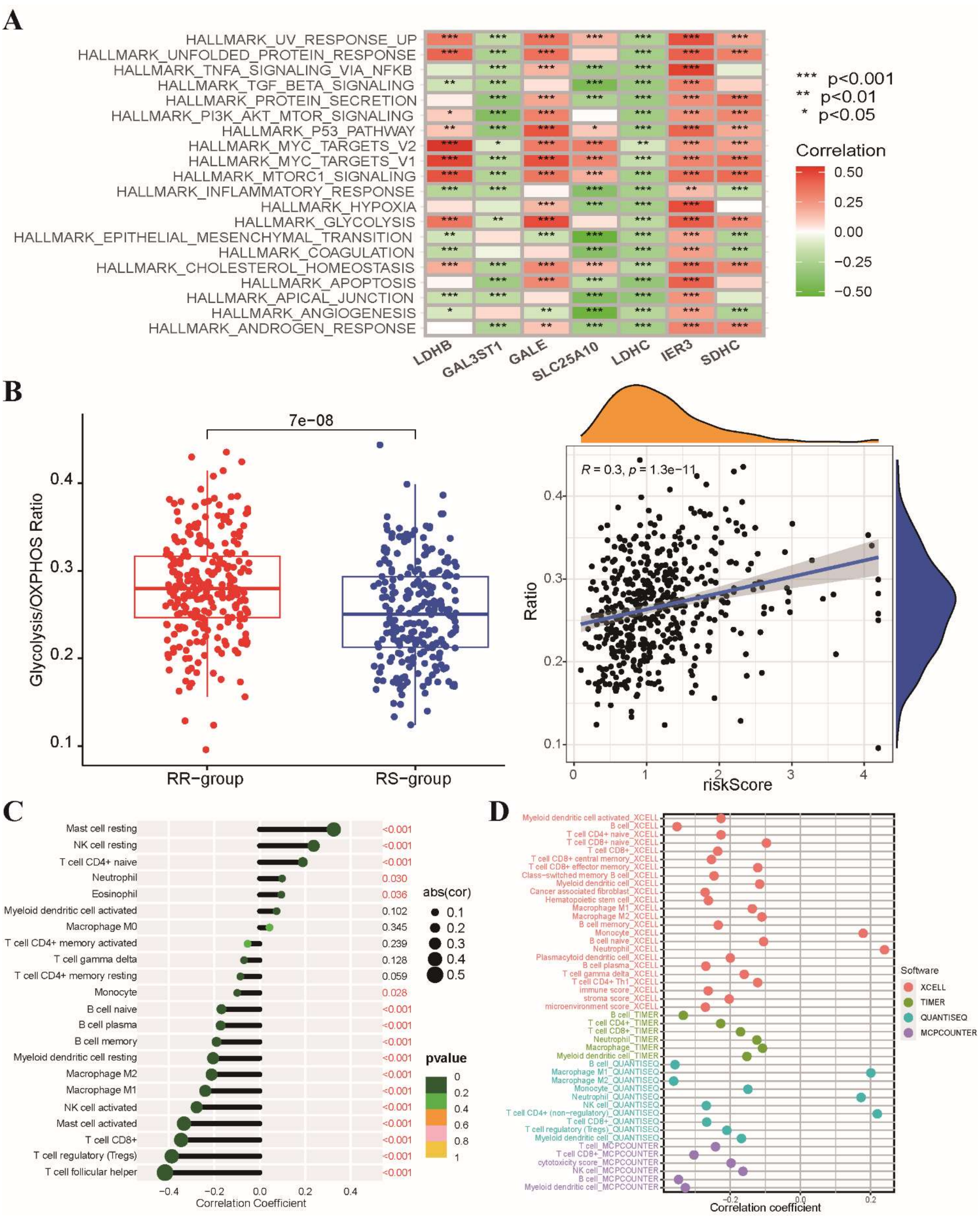

Figure S4 **(A)** Correlation matrix displaying the relationships between the expression levels of the seven genes in the radiosensitivity index and the activity of key hallmark molecular pathways. **(B)** The balance between glycolysis and OXPHOS in relation to radiotherapy response. **(C)** Correlation matrix showcasing the associations between various immune cell subpopulations and the radiosensitivity index. **(D)** Comparative analysis assessing CD8 T cell infiltration levels between RS and RR groups, evaluated using four separate algorithms: XCELL, TIMER, QUANTISEQ, and MCPCOUNTER. * p<0.05, ** p<0.01, *** p<0.001.

Table S1 | Sequences of the primer used for qRT-PCR

mRNA	Forward primer	Reverse primer
SLC25A10	GCAGACTTGGTCAACGTCAGGA	CATGGTTGCACCCGAGAACAGT
SDHC	GGTTCAAACCGTCCTCTGTCTC	CGACATGCCAAAAAGAGAGACCC
LDHC	TTGGAACTGGTGCCGTAGGCAT	GACTGCCATGCTGAAGATCCATC
LDHB	GGACAAGTTGGTATGGCGTGTG	AAGCTCCCATGCTGCAGATCCA
IER3	GCAGCCGCAGGGTTCTCTACC	CTCTTCAGCCATCAGGATCTGG
GALE	AGGTGATGTGGCAGCCTGTTAC	TTGCGTGCCAAAGCCTGAAGGA
GAL3ST1	TGCTGGTGTACTCCTATGCCGT	GGATCACTGCCTCTGGCTCGA
GAPDH	GTCTCCTCTGACTTCAACAGCG	ACCACCCTGTTGCTGTAGCCAA
